# How a Clergymen Cured His Appetite for Tobacco

**Published:** 1864-12

**Authors:** 


How a Clergymen Cured his Appetite for Tobacco.—I had a deep well of very cold water, and whenever the evil appetite craved indulgence, I resorted immediately to fresh drawn water. Of this I drank what I desired, and then continued to hold water in my mouth, throwing out and taking in successive mouthfulls, until the craving ceased. By a faithful adherence to this practice for about a month, *I was cured:* and from that time to this have been entirely free from any appetite for tobacco.

